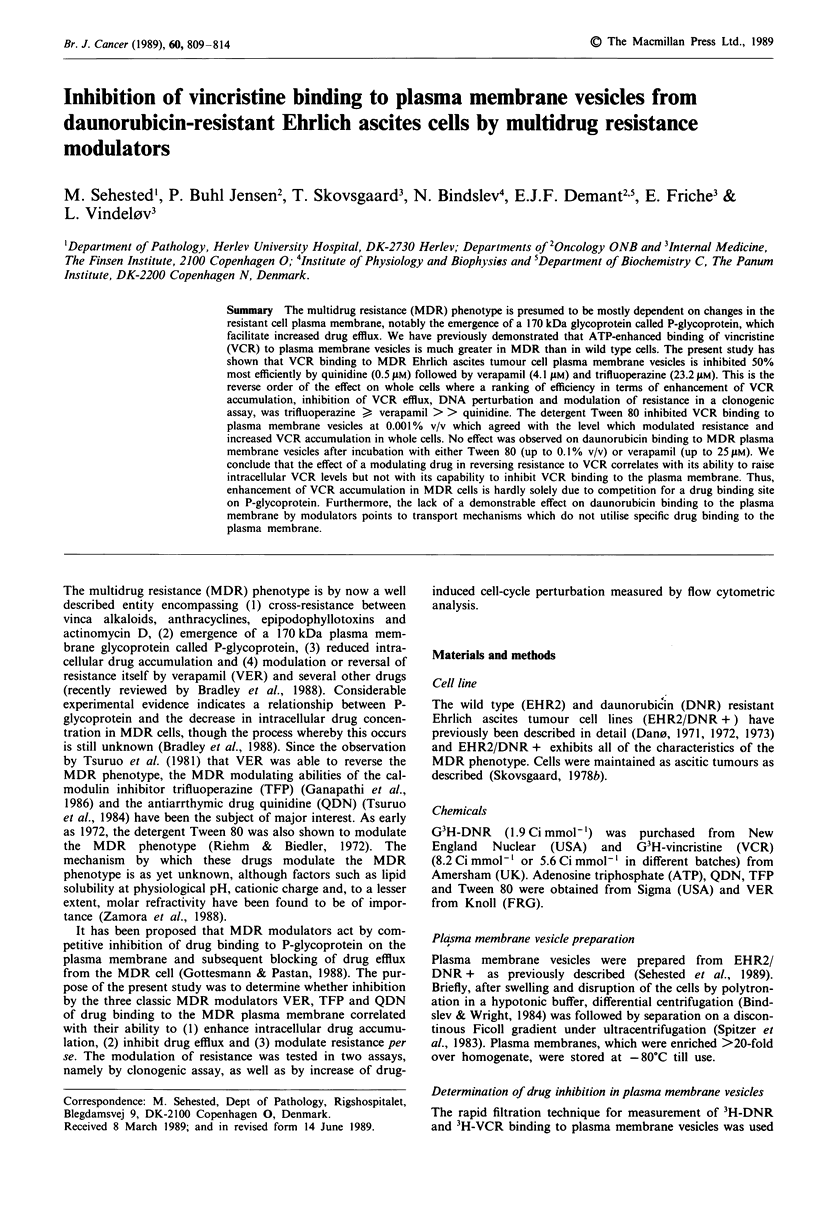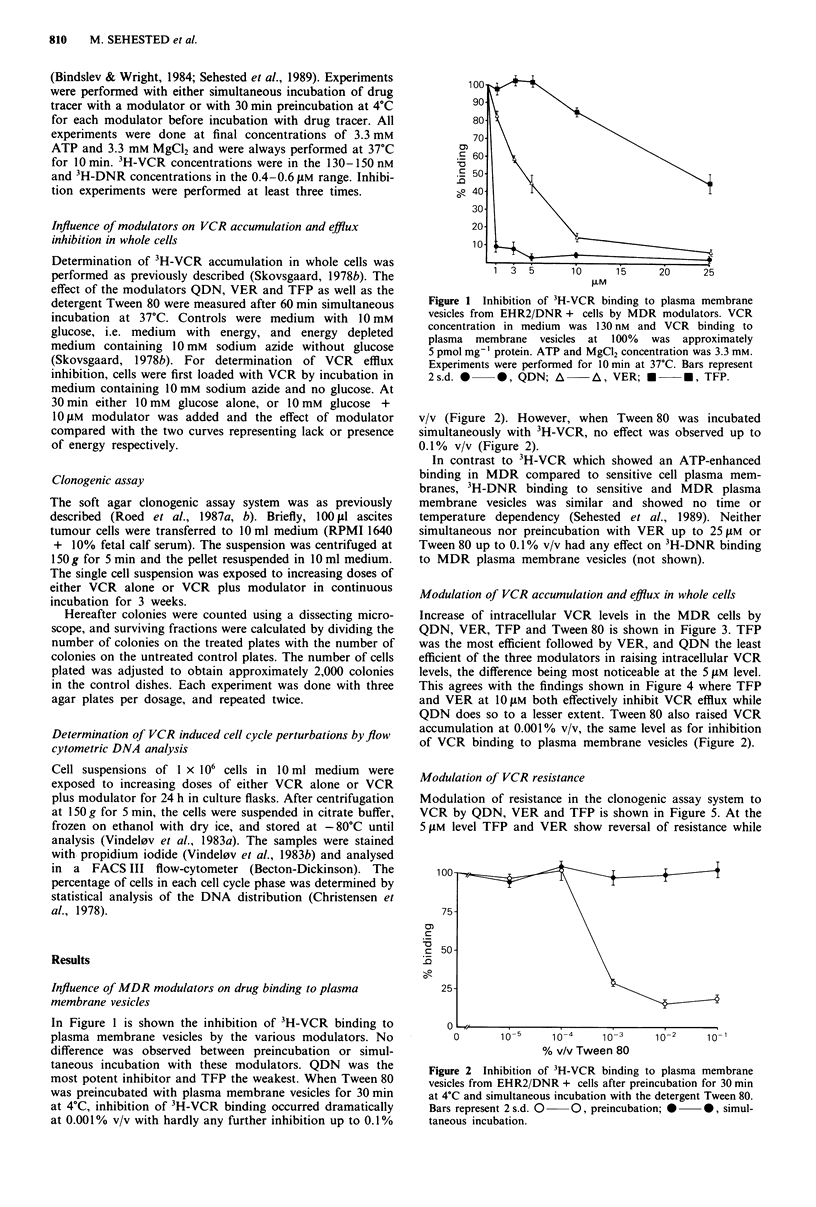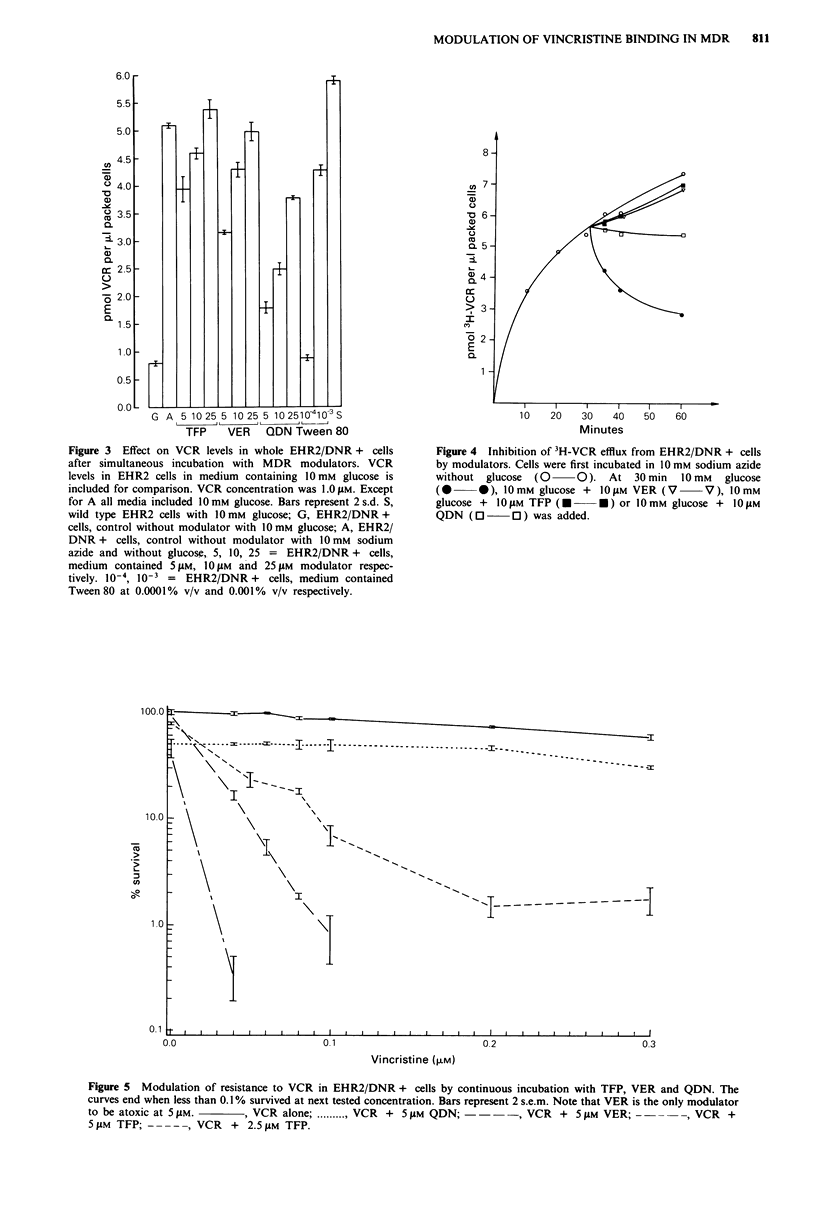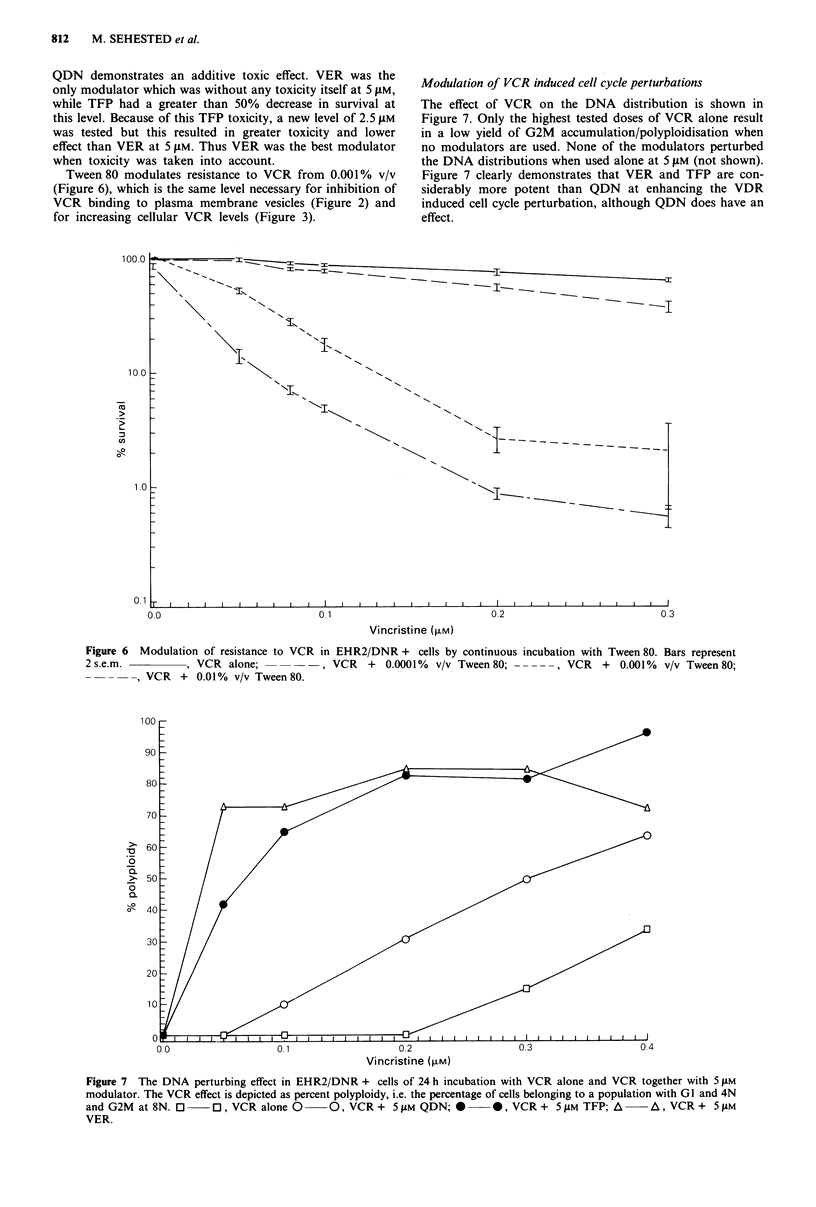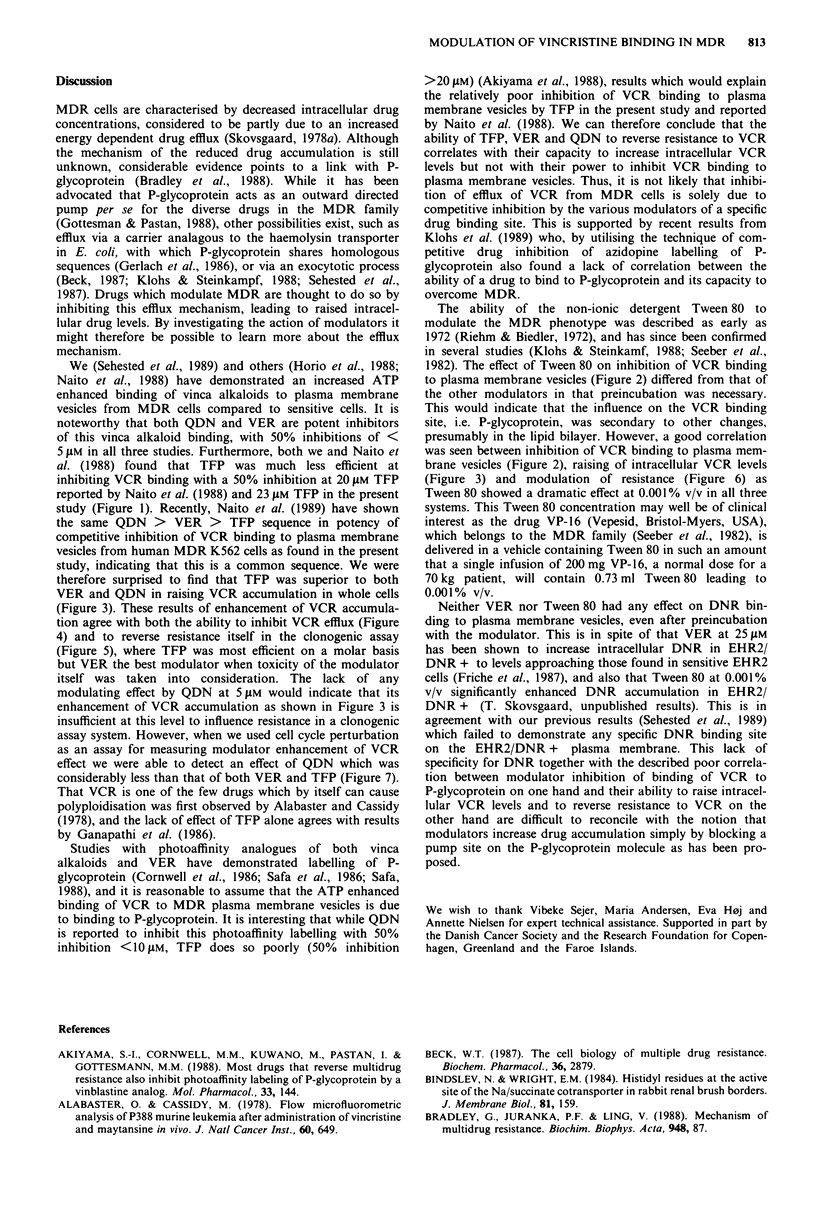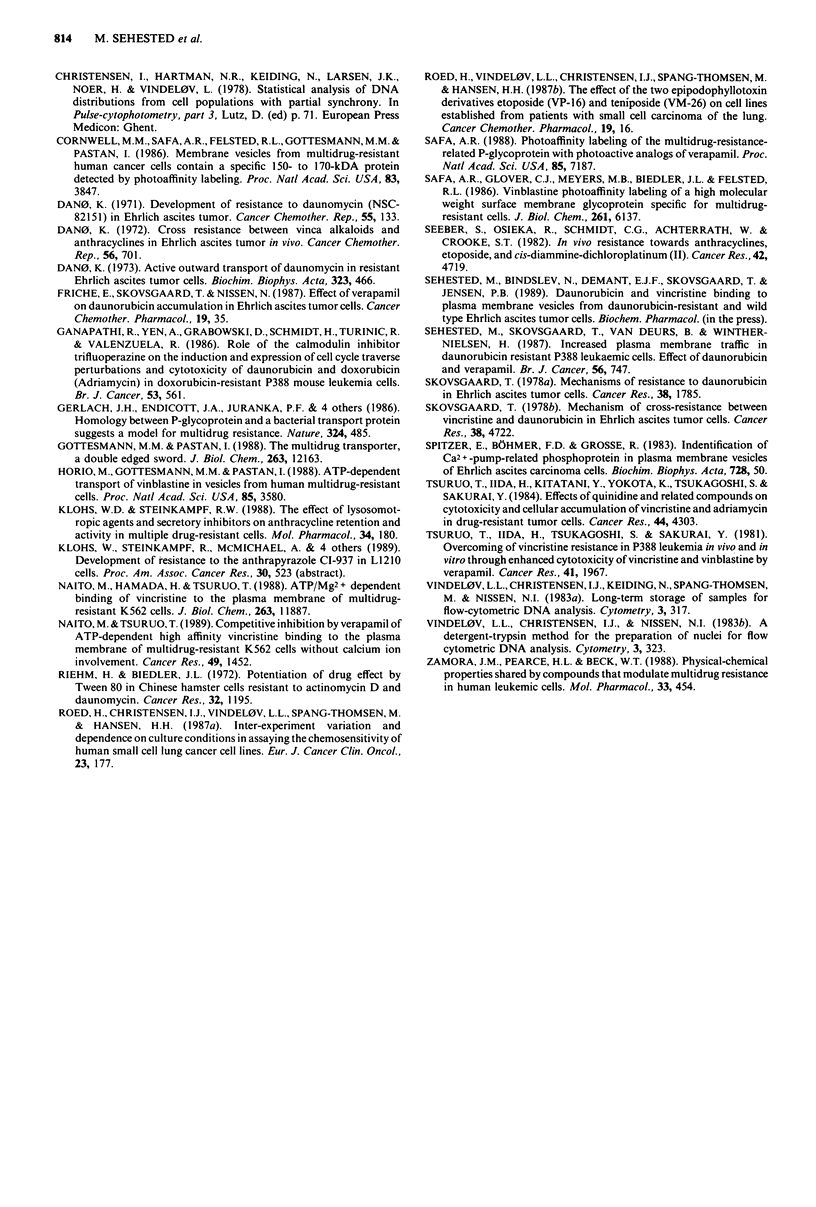# Inhibition of vincristine binding to plasma membrane vesicles from daunorubicin-resistant Ehrlich ascites cells by multidrug resistance modulators.

**DOI:** 10.1038/bjc.1989.371

**Published:** 1989-12

**Authors:** M. Sehested, P. B. Jensen, T. Skovsgaard, N. Bindslev, E. J. Demant, E. Friche, L. VindelÃ¸v

**Affiliations:** Department of Pathology, Herlev University Hospital, Denmark.

## Abstract

The multidrug resistance (MDR) phenotype is presumed to be mostly dependent on changes in the resistant cell plasma membrane, notably the emergence of a 170 kDa glycoprotein called P-glycoprotein, which facilitate increased drug efflux. We have previously demonstrated that ATP-enhanced binding of vincristine (VCR) to plasma membrane vesicles is much greater in MDR than in wild type cells. The present study has shown that VCR binding to MDR Ehrlich ascites tumour cell plasma membrane vesicles is inhibited 50% most efficiently by quinidine (0.5 microM) followed by verapamil (4.1 microM) and trifluoperazine (23.2 microM). This is the reverse order of the effect on whole cells where a ranking of efficiency in terms of enhancement of VCR accumulation, inhibition of VCR efflux, DNA perturbation and modulation of resistance in a clonogenic assay, was trifluoperazine greater than or equal to verapamil much greater than quinidine. The detergent Tween 80 inhibited VCR binding to plasma membrane vesicles at 0.001% v/v which agreed with the level which modulated resistance and increased VCR accumulation in whole cells. No effect was observed on daunorubicin binding to MDR plasma membrane vesicles after incubation with either Tween 80 (up to 0.1% v/v) or verapamil (up to 25 microM). We conclude that the effect of a modulating drug in reversing resistance to VCR correlates with its ability to raise intracellular VCR levels but not with its capability to inhibit VCR binding to the plasma membrane. Thus, enhancement of VCR accumulation in MDR cells is hardly solely due to competition for a drug binding site on P-glycoprotein. Furthermore, the lack of a demonstrable effect on daunorubicin binding to the plasma membrane by modulators points to transport mechanisms which do not utilise specific drug binding to the plasma membrane.


Br. J. Cancer (1989), 60, 809-814
© The Macmillan Press Ltd., 1989

### Inhibition of vincristine binding to plasma membrane vesicles from daunorubicin-resistant Ehrlich ascites cells by multidrug resistance modulators

M. Sehested[1], P. Buhl Jensen[2], T. Skovsgaard[3], N. Bindslev[4], E.J.F. Demant[2,5], E. Friche[3] & L. Vindeløv[3]

[1]*Department of Pathology, Herlev University Hospital, DK-2730 Herlev; Departments of* [2]*Oncology ONB and* [3]*Internal Medicine, The Finsen Institute, 2100 Copenhagen O;* [4]*Institute of Physiology and Biophysics and* [5]*Department of Biochemistry C, The Panum Institute, DK-2200 Copenhagen N, Denmark.*

**Summary** The multidrug resistance (MDR) phenotype is presumed to be mostly dependent on changes in the resistant cell plasma membrane, notably the emergence of a 170 kDa glycoprotein called P-glycoprotein, which facilitate increased drug efflux. We have previously demonstrated that ATP-enhanced binding of vincristine (VCR) to plasma membrane vesicles is much greater in MDR than in wild type cells. The present study has shown that VCR binding to MDR Ehrlich ascites tumour cell plasma membrane vesicles is inhibited 50% most efficiently by quinidine (0.5 μM) followed by verapamil (4.1 μM) and trifluoperazine (23.2 μM). This is the reverse order of the effect on whole cells where a ranking of efficiency in terms of enhancement of VCR accumulation, inhibition of VCR efflux, DNA perturbation and modulation of resistance in a clonogenic assay, was trifluoperazine $\geqslant$ verapamil $>>$ quinidine. The detergent Tween 80 inhibited VCR binding to plasma membrane vesicles at 0.001% v/v which agreed with the level which modulated resistance and increased VCR accumulation in whole cells. No effect was observed on daunorubicin binding to MDR plasma membrane vesicles after incubation with either Tween 80 (up to 0.1% v/v) or verapamil (up to 25 μM). We conclude that the effect of a modulating drug in reversing resistance to VCR correlates with its ability to raise intracellular VCR levels but not with its capability to inhibit VCR binding to the plasma membrane. Thus, enhancement of VCR accumulation in MDR cells is hardly solely due to competition for a drug binding site on P-glycoprotein. Furthermore, the lack of a demonstrable effect on daunorubicin binding to the plasma membrane by modulators points to transport mechanisms which do not utilise specific drug binding to the plasma membrane.

The multidrug resistance (MDR) phenotype is by now a well described entity encompassing (1) cross-resistance between vinca alkaloids, anthracyclines, epipodophyllotoxins and actinomycin D, (2) emergence of a 170 kDa plasma membrane glycoprotein called P-glycoprotein, (3) reduced intracellular drug accumulation and (4) modulation or reversal of resistance itself by verapamil (VER) and several other drugs (recently reviewed by Bradley *et al.*, 1988). Considerable experimental evidence indicates a relationship between P-glycoprotein and the decrease in intracellular drug concentration in MDR cells, though the process whereby this occurs is still unknown (Bradley *et al.*, 1988). Since the observation by Tsuruo *et al.* (1981) that VER was able to reverse the MDR phenotype, the MDR modulating abilities of the calmodulin inhibitor trifluoperazine (TFP) (Ganapathi *et al.*, 1986) and the antiarrthymic drug quinidine (QDN) (Tsuruo *et al.*, 1984) have been the subject of major interest. As early as 1972, the detergent Tween 80 was also shown to modulate the MDR phenotype (Riehm & Biedler, 1972). The mechanism by which these drugs modulate the MDR phenotype is as yet unknown, although factors such as lipid solubility at physiological pH, cationic charge and, to a lesser extent, molar refractivity have been found to be of importance (Zamora *et al.*, 1988).

It has been proposed that MDR modulators act by competitive inhibition of drug binding to P-glycoprotein on the plasma membrane and subsequent blocking of drug efflux from the MDR cell (Gottesmann & Pastan, 1988). The purpose of the present study was to determine whether inhibition by the three classic MDR modulators VER, TFP and QDN of drug binding to the MDR plasma membrane correlated with their ability to (1) enhance intracellular drug accumulation, (2) inhibit drug efflux and (3) modulate resistance *per se*. The modulation of resistance was tested in two assays, namely by clonogenic assay, as well as by increase of drug-induced cell-cycle perturbation measured by flow cytometric analysis.

Correspondence: M. Sehested, Dept of Pathology, Rigshospitalet, Blegdamsvej 9, DK-2100 Copenhagen O, Denmark.
Received 8 March 1989; and in revised form 14 June 1989.

### Materials and methods

### Cell line

The wild type (EHR2) and daunorubicin (DNR) resistant Ehrlich ascites tumour cell lines (EHR2/DNR+) have previously been described in detail (Danø, 1971, 1972, 1973) and EHR2/DNR+ exhibits all of the characteristics of the MDR phenotype. Cells were maintained as ascitic tumours as described (Skovsgaard, 1978*b*).

### Chemicals

G$^3$H-DNR (1.9 Ci mmol$^{-1}$) was purchased from New England Nuclear (USA) and G$^3$H-vincristine (VCR) (8.2 Ci mmol$^{-1}$ or 5.6 Ci mmol$^{-1}$ in different batches) from Amersham (UK). Adenosine triphosphate (ATP), QDN, TFP and Tween 80 were obtained from Sigma (USA) and VER from Knoll (FRG).

### Plasma membrane vesicle preparation

Plasma membrane vesicles were prepared from EHR2/DNR+ as previously described (Sehested *et al.*, 1989). Briefly, after swelling and disruption of the cells by polytronation in a hypotonic buffer, differential centrifugation (Bindslev & Wright, 1984) was followed by separation on a discontinous Ficoll gradient under ultracentrifugation (Spitzer *et al.*, 1983). Plasma membranes, which were enriched >20-fold over homogenate, were stored at −80°C till use.

### Determination of drug inhibition in plasma membrane vesicles

The rapid filtration technique for measurement of $^3$H-DNR and $^3$H-VCR binding to plasma membrane vesicles was used

(Bindslev & Wright, 1984; Sehested *et al.*, 1989). Experiments were performed with either simultaneous incubation of drug tracer with a modulator or with 30 min preincubation at 4°C for each modulator before incubation with drug tracer. All experiments were done at final concentrations of 3.3 mM ATP and 3.3 mM $MgCl_2$ and were always performed at 37°C for 10 min. $^3$H-VCR concentrations were in the 130–150 nM and $^3$H-DNR concentrations in the 0.4–0.6 μM range. Inhibition experiments were performed at least three times.

*Influence of modulators on VCR accumulation and efflux inhibition in whole cells*

Determination of $^3$H-VCR accumulation in whole cells was performed as previously described (Skovsgaard, 1978*b*). The effect of the modulators QDN, VER and TFP as well as the detergent Tween 80 were measured after 60 min simultaneous incubation at 37°C. Controls were medium with 10 mM glucose, i.e. medium with energy, and energy depleted medium containing 10 mM sodium azide without glucose (Skovsgaard, 1978*b*). For determination of VCR efflux inhibition, cells were first loaded with VCR by incubation in medium containing 10 mM sodium azide and no glucose. At 30 min either 10 mM glucose alone, or 10 mM glucose + 10 μM modulator was added and the effect of modulator compared with the two curves representing lack or presence of energy respectively.

*Clonogenic assay*

The soft agar clonogenic assay system was as previously described (Roed *et al.*, 1987*a*, *b*). Briefly, 100 μl ascites tumour cells were transferred to 10 ml medium (RPMI 1640 + 10% fetal calf serum). The suspension was centrifuged at 150 *g* for 5 min and the pellet resuspended in 10 ml medium. The single cell suspension was exposed to increasing doses of either VCR alone or VCR plus modulator in continuous incubation for 3 weeks.

Hereafter colonies were counted using a dissecting microscope, and surviving fractions were calculated by dividing the number of colonies on the treated plates with the number of colonies on the untreated control plates. The number of cells plated was adjusted to obtain approximately 2,000 colonies in the control dishes. Each experiment was done with three agar plates per dosage, and repeated twice.

*Determination of VCR induced cell cycle perturbations by flow cytometric DNA analysis*

Cell suspensions of $1 \times 10^6$ cells in 10 ml medium were exposed to increasing doses of either VCR alone or VCR plus modulator for 24 h in culture flasks. After centrifugation at 150 *g* for 5 min, the cells were suspended in citrate buffer, frozen on ethanol with dry ice, and stored at −80°C until analysis (Vindeløv *et al.*, 1983*a*). The samples were stained with propidium iodide (Vindeløv *et al.*, 1983*b*) and analysed in a FACS III flow-cytometer (Becton-Dickinson). The percentage of cells in each cell cycle phase was determined by statistical analysis of the DNA distribution (Christensen *et al.*, 1978).

### Results

*Influence of MDR modulators on drug binding to plasma membrane vesicles*

In Figure 1 is shown the inhibition of $^3$H-VCR binding to plasma membrane vesicles by the various modulators. No difference was observed between preincubation or simultaneous incubation with these modulators. QDN was the most potent inhibitor and TFP the weakest. When Tween 80 was preincubated with plasma membrane vesicles for 30 min at 4°C, inhibition of $^3$H-VCR binding occurred dramatically at 0.001% v/v with hardly any further inhibition up to 0.1%

**Figure 1** Inhibition of $^3$H-VCR binding to plasma membrane vesicles from EHR2/DNR + cells by MDR modulators. VCR concentration in medium was 130 nM and VCR binding to plasma membrane vesicles at 100% was approximately 5 pmol $mg^{-1}$ protein. ATP and $MgCl_2$ concentration was 3.3 mM. Experiments were performed for 10 min at 37°C. Bars represent 2 s.d. ●——●, QDN; △——△, VER; ■——■, TFP.

v/v (Figure 2). However, when Tween 80 was incubated simultaneously with $^3$H-VCR, no effect was observed up to 0.1% v/v (Figure 2).

In contrast to $^3$H-VCR which showed an ATP-enhanced binding in MDR compared to sensitive cell plasma membranes, $^3$H-DNR binding to sensitive and MDR plasma membrane vesicles was similar and showed no time or temperature dependency (Sehested *et al.*, 1989). Neither simultaneous nor preincubation with VER up to 25 μM or Tween 80 up to 0.1% v/v had any effect on $^3$H-DNR binding to MDR plasma membrane vesicles (not shown).

*Modulation of VCR accumulation and efflux in whole cells*

Increase of intracellular VCR levels in the MDR cells by QDN, VER, TFP and Tween 80 is shown in Figure 3. TFP was the most efficient followed by VER, and QDN the least efficient of the three modulators in raising intracellular VCR levels, the difference being most noticeable at the 5 μM level. This agrees with the findings shown in Figure 4 where TFP and VER at 10 μM both effectively inhibit VCR efflux while QDN does so to a lesser extent. Tween 80 also raised VCR accumulation at 0.001% v/v, the same level as for inhibition of VCR binding to plasma membrane vesicles (Figure 2).

*Modulation of VCR resistance*

Modulation of resistance in the clonogenic assay system to VCR by QDN, VER and TFP is shown in Figure 5. At the 5 μM level TFP and VER show reversal of resistance while

**Figure 2** Inhibition of $^3$H-VCR binding to plasma membrane vesicles from EHR2/DNR + cells after preincubation for 30 min at 4°C and simultaneous incubation with the detergent Tween 80. Bars represent 2 s.d. ○——○, preincubation; ●——●, simultaneous incubation.

**Figure 3** Effect on VCR levels in whole EHR2/DNR + cells after simultaneous incubation with MDR modulators. VCR levels in EHR2 cells in medium containing 10 mM glucose is included for comparison. VCR concentration was 1.0 μM. Except for A all media included 10 mM glucose. Bars represent 2 s.d. S, wild type EHR2 cells with 10 mM glucose; G, EHR2/DNR + cells, control without modulator with 10 mM glucose; A, EHR2/DNR + cells, control without modulator with 10 mM sodium azide and without glucose, 5, 10, 25 = EHR2/DNR + cells, medium contained 5 μM, 10 μM and 25 μM modulator respectively. $10^{-4}$, $10^{-3}$ = EHR2/DNR + cells, medium contained Tween 80 at 0.0001% v/v and 0.001% v/v respectively.

**Figure 4** Inhibition of $^3$H-VCR efflux from EHR2/DNR + cells by modulators. Cells were first incubated in 10 mM sodium azide without glucose (○——○). At 30 min 10 mM glucose (●——●), 10 mM glucose + 10 μM VER (▽——▽), 10 mM glucose + 10 μM TFP (■——■) or 10 mM glucose + 10 μM QDN (□——□) was added.

**Figure 5** Modulation of resistance to VCR in EHR2/DNR + cells by continuous incubation with TFP, VER and QDN. The curves end when less than 0.1% survived at next tested concentration. Bars represent 2 s.e.m. Note that VER is the only modulator to be atoxic at 5 μM. ————, VCR alone; ........, VCR + 5 μM QDN; — — — —, VCR + 5 μM VER; – – – – – –, VCR + 5 μM TFP; - - - - -, VCR + 2.5 μM TFP.

QDN demonstrates an additive toxic effect. VER was the only modulator which was without any toxicity itself at 5 μM, while TFP had a greater than 50% decrease in survival at this level. Because of this TFP toxicity, a new level of 2.5 μM was tested but this resulted in greater toxicity and lower effect than VER at 5 μM. Thus VER was the best modulator when toxicity was taken into account.

Tween 80 modulates resistance to VCR from 0.001% v/v (Figure 6), which is the same level necessary for inhibition of VCR binding to plasma membrane vesicles (Figure 2) and for increasing cellular VCR levels (Figure 3).

### *Modulation of VCR induced cell cycle perturbations*

The effect of VCR on the DNA distribution is shown in Figure 7. Only the highest tested doses of VCR alone result in a low yield of G2M accumulation/polyploidisation when no modulators are used. None of the modulators perturbed the DNA distributions when used alone at 5 μM (not shown). Figure 7 clearly demonstrates that VER and TFP are considerably more potent than QDN at enhancing the VDR induced cell cycle perturbation, although QDN does have an effect.

**Figure 6** Modulation of resistance to VCR in EHR2/DNR+ cells by continuous incubation with Tween 80. Bars represent 2 s.e.m. ———, VCR alone; — — — —, VCR + 0.0001% v/v Tween 80; - - - - -, VCR + 0.001% v/v Tween 80; — - — - —, VCR + 0.01% v/v Tween 80.

**Figure 7** The DNA perturbing effect in EHR2/DNR+ cells of 24 h incubation with VCR alone and VCR together with 5 μM modulator. The VCR effect is depicted as percent polyploidy, i.e. the percentage of cells belonging to a population with G1 and 4N and G2M at 8N. □——□, VCR alone ○——○, VCR + 5 μM QDN; ●——●, VCR + 5 μM TFP; △——△, VCR + 5 μM VER.

### Discussion

MDR cells are characterised by decreased intracellular drug concentrations, considered to be partly due to an increased energy dependent drug efflux (Skovsgaard, 1978*a*). Although the mechanism of the reduced drug accumulation is still unknown, considerable evidence points to a link with P-glycoprotein (Bradley *et al.*, 1988). While it has been advocated that P-glycoprotein acts as an outward directed pump *per se* for the diverse drugs in the MDR family (Gottesman & Pastan, 1988), other possibilities exist, such as efflux via a carrier analagous to the haemolysin transporter in *E. coli*, with which P-glycoprotein shares homologous sequences (Gerlach *et al.*, 1986), or via an exocytotic process (Beck, 1987; Klohs & Steinkampf, 1988; Sehested *et al.*, 1987). Drugs which modulate MDR are thought to do so by inhibiting this efflux mechanism, leading to raised intracellular drug levels. By investigating the action of modulators it might therefore be possible to learn more about the efflux mechanism.

We (Sehested *et al.*, 1989) and others (Horio *et al.*, 1988; Naito *et al.*, 1988) have demonstrated an increased ATP enhanced binding of vinca alkaloids to plasma membrane vesicles from MDR cells compared to sensitive cells. It is noteworthy that both QDN and VER are potent inhibitors of this vinca alkaloid binding, with 50% inhibitions of $<$ 5 μM in all three studies. Furthermore, both we and Naito *et al.* (1988) found that TFP was much less efficient at inhibiting VCR binding with a 50% inhibition at 20 μM TFP reported by Naito *et al.* (1988) and 23 μM TFP in the present study (Figure 1). Recently, Naito *et al.* (1989) have shown the same QDN $>$ VER $>$ TFP sequence in potency of competitive inhibition of VCR binding to plasma membrane vesicles from human MDR K562 cells as found in the present study, indicating that this is a common sequence. We were therefore surprised to find that TFP was superior to both VER and QDN in raising VCR accumulation in whole cells (Figure 3). These results of enhancement of VCR accumulation agree with both the ability to inhibit VCR efflux (Figure 4) and to reverse resistance itself in the clonogenic assay (Figure 5), where TFP was most efficient on a molar basis but VER the best modulator when toxicity of the modulator itself was taken into consideration. The lack of any modulating effect by QDN at 5 μM would indicate that its enhancement of VCR accumulation as shown in Figure 3 is insufficient at this level to influence resistance in a clonogenic assay system. However, when we used cell cycle perturbation as an assay for measuring modulator enhancement of VCR effect we were able to detect an effect of QDN which was considerably less than that of both VER and TFP (Figure 7). That VCR is one of the few drugs which by itself can cause polyploidisation was first observed by Alabaster and Cassidy (1978), and the lack of effect of TFP alone agrees with results by Ganapathi *et al.* (1986).

Studies with photoaffinity analogues of both vinca alkaloids and VER have demonstrated labelling of P-glycoprotein (Cornwell *et al.*, 1986; Safa *et al.*, 1986; Safa, 1988), and it is reasonable to assume that the ATP enhanced binding of VCR to MDR plasma membrane vesicles is due to binding to P-glycoprotein. It is interesting that while QDN is reported to inhibit this photoaffinity labelling with 50% inhibition $<$10 μM, TFP does so poorly (50% inhibition $>$20 μM) (Akiyama *et al.*, 1988), results which would explain the relatively poor inhibition of VCR binding to plasma membrane vesicles by TFP in the present study and reported by Naito *et al.* (1988). We can therefore conclude that the ability of TFP, VER and QDN to reverse resistance to VCR correlates with their capacity to increase intracellular VCR levels but not with their power to inhibit VCR binding to plasma membrane vesicles. Thus, it is not likely that inhibition of efflux of VCR from MDR cells is solely due to competitive inhibition by the various modulators of a specific drug binding site. This is supported by recent results from Klohs *et al.* (1989) who, by utilising the technique of competitive drug inhibition of azidopine labelling of P-glycoprotein also found a lack of correlation between the ability of a drug to bind to P-glycoprotein and its capacity to overcome MDR.

The ability of the non-ionic detergent Tween 80 to modulate the MDR phenotype was described as early as 1972 (Riehm & Biedler, 1972), and has since been confirmed in several studies (Klohs & Steinkamf, 1988; Seeber *et al.*, 1982). The effect of Tween 80 on inhibition of VCR binding to plasma membrane vesicles (Figure 2) differed from that of the other modulators in that preincubation was necessary. This would indicate that the influence on the VCR binding site, i.e. P-glycoprotein, was secondary to other changes, presumably in the lipid bilayer. However, a good correlation was seen between inhibition of VCR binding to plasma membrane vesicles (Figure 2), raising of intracellular VCR levels (Figure 3) and modulation of resistance (Figure 6) as Tween 80 showed a dramatic effect at 0.001% v/v in all three systems. This Tween 80 concentration may well be of clinical interest as the drug VP-16 (Vepesid, Bristol-Myers, USA), which belongs to the MDR family (Seeber *et al.*, 1982), is delivered in a vehicle containing Tween 80 in such an amount that a single infusion of 200 mg VP-16, a normal dose for a 70 kg patient, will contain 0.73 ml Tween 80 leading to 0.001% v/v.

Neither VER nor Tween 80 had any effect on DNR binding to plasma membrane vesicles, even after preincubation with the modulator. This is in spite of that VER at 25 μM has been shown to increase intracellular DNR in EHR2/DNR+ to levels approaching those found in sensitive EHR2 cells (Friche *et al.*, 1987), and also that Tween 80 at 0.001% v/v significantly enhanced DNR accumulation in EHR2/DNR+ (T. Skovsgaard, unpublished results). This is in agreement with our previous results (Sehested *et al.*, 1989) which failed to demonstrate any specific DNR binding site on the EHR2/DNR+ plasma membrane. This lack of specificity for DNR together with the described poor correlation between modulator inhibition of binding of VCR to P-glycoprotein on one hand and their ability to raise intracellular VCR levels and to reverse resistance to VCR on the other hand are difficult to reconcile with the notion that modulators increase drug accumulation simply by blocking a pump site on the P-glycoprotein molecule as has been proposed.

We wish to thank Vibeke Sejer, Maria Andersen, Eva Høj and Annette Nielsen for expert technical assistance. Supported in part by the Danish Cancer Society and the Research Foundation for Copenhagen, Greenland and the Faroe Islands.

### References

AKIYAMA, S.-I., CORNWELL, M.M., KUWANO, M., PASTAN, I. & GOTTESMANN, M.M. (1988). Most drugs that reverse multidrug resistance also inhibit photoaffinity labeling of P-glycoprotein by a vinblastine analog. *Mol. Pharmacol.*, **33**, 144.

ALABASTER, O. & CASSIDY, M. (1978). Flow microfluorometric analysis of P388 murine leukemia after administration of vincristine and maytansine *in vivo*. *J. Natl Cancer Inst.*, **60**, 649.

BECK, W.T. (1987). The cell biology of multiple drug resistance. *Biochem. Pharmacol.*, **36**, 2879.

BINDSLEV, N. & WRIGHT, E.M. (1984). Histidyl residues at the active site of the Na/succinate cotransporter in rabbit renal brush borders. *J. Membrane Biol.*, **81**, 159.

BRADLEY, G., JURANKA, P.F. & LING, V. (1988). Mechanism of multidrug resistance. *Biochim. Biophys. Acta*, **948**, 87.

CHRISTENSEN, I., HARTMAN, N.R., KEIDING, N., LARSEN, J.K., NOER, H. & VINDELØV, L. (1978). Statistical analysis of DNA distributions from cell populations with partial synchrony. In *Pulse-cytophotometry, part 3*, Lutz, D. (ed) p. 71. European Press Medicon: Ghent.

CORNWELL, M.M., SAFA, A.R., FELSTED, R.L., GOTTESMANN, M.M. & PASTAN, I. (1986). Membrane vesicles from multidrug-resistant human cancer cells contain a specific 150- to 170-kDA protein detected by photoaffinity labeling. *Proc. Natl Acad. Sci. USA*, **83**, 3847.

DANØ, K. (1971). Development of resistance to daunomycin (NSC-82151) in Ehrlich ascites tumor. *Cancer Chemother. Rep.*, **55**, 133.

DANØ, K. (1972). Cross resistance between vinca alkaloids and anthracyclines in Ehrlich ascites tumor *in vivo*. *Cancer Chemother. Rep.*, **56**, 701.

DANØ, K. (1973). Active outward transport of daunomycin in resistant Ehrlich ascites tumor cells. *Biochim. Biophys. Acta*, **323**, 466.

FRICHE, E., SKOVSGAARD, T. & NISSEN, N. (1987). Effect of verapamil on daunorubicin accumulation in Ehrlich ascites tumor cells. *Cancer Chemother. Pharmacol.*, **19**, 35.

GANAPATHI, R., YEN, A., GRABOWSKI, D., SCHMIDT, H., TURINIC, R. & VALENZUELA, R. (1986). Role of the calmodulin inhibitor trifluoperazine on the induction and expression of cell cycle traverse perturbations and cytotoxicity of daunorubicin and doxorubicin (Adriamycin) in doxorubicin-resistant P388 mouse leukemia cells. *Br. J. Cancer*, **53**, 561.

GERLACH, J.H., ENDICOTT, J.A., JURANKA, P.F. & 4 others (1986). Homology between P-glycoprotein and a bacterial transport protein suggests a model for multidrug resistance. *Nature*, **324**, 485.

GOTTESMANN, M.M. & PASTAN, I. (1988). The multidrug transporter, a double edged sword. *J. Biol. Chem.*, **263**, 12163.

HORIO, M., GOTTESMANN, M.M. & PASTAN, I. (1988). ATP-dependent transport of vinblastine in vesicles from human multidrug-resistant cells. *Proc. Natl Acad. Sci. USA*, **85**, 3580.

KLOHS, W.D. & STEINKAMPF, R.W. (1988). The effect of lysosomotropic agents and secretory inhibitors on anthracycline retention and activity in multiple drug-resistant cells. *Mol. Pharmacol.*, **34**, 180.

KLOHS, W., STEINKAMPF, R., MCMICHAEL, A. & 4 others (1989). Development of resistance to the anthrapyrazole CI-937 in L1210 cells. *Proc. Am. Assoc. Cancer Res.*, **30**, 523 (abstract).

NAITO, M., HAMADA, H. & TSURUO, T. (1988). ATP/$Mg^{2+}$ dependent binding of vincristine to the plasma membrane of multidrug-resistant K562 cells. *J. Biol. Chem.*, **263**, 11887.

NAITO, M. & TSURUO, T. (1989). Competitive inhibition by verapamil of ATP-dependent high affinity vincristine binding to the plasma membrane of multidrug-resistant K562 cells without calcium ion involvement. *Cancer Res.*, **49**, 1452.

RIEHM, H. & BIEDLER, J.L. (1972). Potentiation of drug effect by Tween 80 in Chinese hamster cells resistant to actinomycin D and daunomycin. *Cancer Res.*, **32**, 1195.

ROED, H., CHRISTENSEN, I.J., VINDELØV, L.L., SPANG-THOMSEN, M. & HANSEN, H.H. (1987*a*). Inter-experiment variation and dependence on culture conditions in assaying the chemosensitivity of human small cell lung cancer cell lines. *Eur. J. Cancer Clin. Oncol.*, **23**, 177.

ROED, H., VINDELØV, L.L., CHRISTENSEN, I.J., SPANG-THOMSEN, M. & HANSEN, H.H. (1987*b*). The effect of the two epipodophyllotoxin derivatives etoposide (VP-16) and teniposide (VM-26) on cell lines established from patients with small cell carcinoma of the lung. *Cancer Chemother. Pharmacol.*, **19**, 16.

SAFA, A.R. (1988). Photoaffinity labeling of the multidrug-resistance-related P-glycoprotein with photoactive analogs of verapamil. *Proc. Natl Acad. Sci. USA*, **85**, 7187.

SAFA, A.R., GLOVER, C.J., MEYERS, M.B., BIEDLER, J.L. & FELSTED, R.L. (1986). Vinblastine photoaffinity labeling of a high molecular weight surface membrane glycoprotein specific for multidrug-resistant cells. *J. Biol. Chem.*, **261**, 6137.

SEEBER, S., OSIEKA, R., SCHMIDT, C.G., ACHTERRATH, W. & CROOKE, S.T. (1982). *In vivo* resistance towards anthracyclines, etoposide, and *cis*-diammine-dichloroplatinum (II). *Cancer Res.*, **42**, 4719.

SEHESTED, M., BINDSLEV, N., DEMANT, E.J.F., SKOVSGAARD, T. & JENSEN, P.B. (1989). Daunorubicin and vincristine binding to plasma membrane vesicles from daunorubicin-resistant and wild type Ehrlich ascites tumor cells. *Biochem. Pharmacol.* (in the press).

SEHESTED, M., SKOVSGAARD, T., VAN DEURS, B. & WINTHER-NIELSEN, H. (1987). Increased plasma membrane traffic in daunorubicin resistant P388 leukaemic cells. Effect of daunorubicin and verapamil. *Br. J. Cancer*, **56**, 747.

SKOVSGAARD, T. (1978*a*). Mechanisms of resistance to daunorubicin in Ehrlich ascites tumor cells. *Cancer Res.*, **38**, 1785.

SKOVSGAARD, T. (1978*b*). Mechanism of cross-resistance between vincristine and daunorubicin in Ehrlich ascites tumor cells. *Cancer Res.*, **38**, 4722.

SPITZER, E., BÖHMER, F.D. & GROSSE, R. (1983). Indentification of $Ca^{2+}$-pump-related phosphoprotein in plasma membrane vesicles of Ehrlich ascites carcinoma cells. *Biochim. Biophys. Acta*, **728**, 50.

TSURUO, T., IIDA, H., KITATANI, Y., YOKOTA, K., TSUKAGOSHI, S. & SAKURAI, Y. (1984). Effects of quinidine and related compounds on cytotoxicity and cellular accumulation of vincristine and adriamycin in drug-resistant tumor cells. *Cancer Res.*, **44**, 4303.

TSURUO, T., IIDA, H., TSUKAGOSHI, S. & SAKURAI, Y. (1981). Overcoming of vincristine resistance in P388 leukemia *in vivo* and *in vitro* through enhanced cytotoxicity of vincristine and vinblastine by verapamil. *Cancer Res.*, **41**, 1967.

VINDELØV, L.L., CHRISTENSEN, I.J., KEIDING, N., SPANG-THOMSEN, M. & NISSEN, N.I. (1983*a*). Long-term storage of samples for flow-cytometric DNA analysis. *Cytometry*, **3**, 317.

VINDELØV, L.L., CHRISTENSEN, I.J., & NISSEN, N.I. (1983*b*). A detergent-trypsin method for the preparation of nuclei for flow cytometric DNA analysis. *Cytometry*, **3**, 323.

ZAMORA, J.M., PEARCE, H.L. & BECK, W.T. (1988). Physical-chemical properties shared by compounds that modulate multidrug resistance in human leukemic cells. *Mol. Pharmacol.*, **33**, 454.